# Association of aPTT-Guided Anticoagulation Monitoring with Thromboembolic Events in Patients Receiving V-A ECMO Support: A Systematic Review and Meta-Analysis

**DOI:** 10.3390/jcm12093224

**Published:** 2023-04-30

**Authors:** Sasa Rajsic, Robert Breitkopf, Benedikt Treml, Dragana Jadzic, Christoph Oberleitner, Ulvi Cenk Oezpeker, Nicole Innerhofer, Zoran Bukumiric

**Affiliations:** 1 Department of Anesthesia and Intensive Care Medicine, Medical University Innsbruck, 6020 Innsbruck, Austria; 2 Anesthesia and Intensive Care Department, Pain Therapy Service, Cagliari University, 09042 Cagliari, Italy; 3 Department of Cardiac Surgery, Medical University Innsbruck, 6020 Innsbruck, Austria; 4 Institute of Medical Statistics and Informatics, Faculty of Medicine, University of Belgrade, 11000 Belgrade, Serbia

**Keywords:** activated partial thromboplastin time, aPTT, adverse events, anticoagulation, bleeding, cardiogenic shock, extracorporeal life support, ECMO, monitoring

## Abstract

Background: The initiation of extracorporeal membrane oxygenation (ECMO) is associated with complex inflammatory and coagulatory processes, raising the need for systemic anticoagulation. The balance of anticoagulatory and procoagulant factors is essential, as therapeutic anticoagulation confers a further risk of potentially life-threatening bleeding. Therefore, our study aims to systematize and analyze the most recent evidence regarding anticoagulation monitoring and the thromboembolic events in patients receiving veno-arterial ECMO support. Methods: Using the PRISMA guidelines, we systematically searched the Scopus and PubMed databases up to October 2022. A weighted effects model was employed for the meta-analytic portion of the study. Results: Six studies comprising 1728 patients were included in the final analysis. Unfractionated heparin was used for anticoagulation, with an activated partial thromboplastin time (aPTT) monitoring goal set between 45 and 80 s. The majority of studies aimed to investigate the incidence of adverse events and potential risk factors for thromboembolic and bleeding events. None of the authors found any association of aPTT levels with the occurrence of thromboembolic events. Finally, the most frequent adverse events were hemorrhage (pooled 43%, 95% CI 28.4; 59.5) and any kind of thrombosis (pooled 36%, 95% CI 21.7; 53.7), and more than one-half of patients did not survive to discharge (pooled 54%). Conclusions: Despite the tremendous development of critical care, aPTT-guided systemic anticoagulation is still the standard monitoring tool. We did not find any association of aPTT levels with thrombosis. Further evidence and new trials should clarify the true incidence of thromboembolic events, along with the best anticoagulation and monitoring strategy in veno-arterial ECMO patients.

## 1. Introduction

The initiation of extracorporeal membrane oxygenation (ECMO) is associated with complex inflammatory and coagulatory processes, with the consequent need for systemic anticoagulation [1,2,3,4]. Therapeutic anticoagulation carries an additional risk of potentially life-threatening bleeding [5,6,7]. Therefore, the balance of anticoagulatory and procoagulant factors is essential [3,8,9,10,11].

The most common adverse events during ECMO support are acute kidney injury (51%), bleeding (49%), and thrombosis (22%) [9,12]. However, thrombosis incidence is often underestimated in the literature, most likely due to its clinically asymptomatic course and the retrospective nature of the majority of studies.

The current recommendations on systemic anticoagulation monitoring are based on several in vitro tests, with the main goal of thrombosis and bleeding evasion. The Extracorporeal Life Support Organization (ELSO) guidelines promote the use of activated clotting time (ACT), activated prothrombin time (aPTT), and anti-factor Xa assays, with all the contemplative limitations [4,13,14]. The recommended aPTT goal is set at 1.5–2.5 times the patient’s normal baseline. However, this goal arises from a study conducted more than half a century ago, reporting on a decreased incidence of recurrent venous thromboembolism [15]. This range was never validated in controlled randomized studies or in ECMO patients.

Finally, aPTT goals are set to reduce the risk of thromboembolic events, weighing them against the risk of hemorrhage. Guidelines for systemic anticoagulation during extracorporeal life support, based on strong evidence, are still missing, and systematized information on the relationship of aPTT levels with thrombosis is nonexistent. Multiple authors have strived to analyze the efficacy of aPTT-guided anticoagulation monitoring, but this subject remains controversial [16,17,18,19].

Given the above, we aimed to systematize the evidence and provide a comprehensive analysis of aPTT-guided anticoagulation monitoring and its association with thromboembolic events in V-A ECMO patients. Moreover, we report on potential adverse events and mortality of patients receiving ECMO support.

## 2. Materials and Methods

We performed a systematic review of the literature and a meta-analysis of aPTT-guided anticoagulation monitoring during extracorporeal life support, complying with the PRISMA guidelines (Additional file 1: Table S1) [20]. Our work is registered in the PROSPERO database (CRD42022359465, PROSPERO—an international database of prospectively registered systematic reviews).

The main aim of this work was the systematization of evidence related to the aPTT-guided anticoagulation monitoring strategies. The secondary endpoint included an analysis of a possible relationship between anticoagulation monitoring and thromboembolic events. Therefore, we focused on articles providing information on patients receiving V-A ECMO support, reporting on potential adverse events, and on anticoagulation monitoring. Detailed PICOS criteria for inclusion and exclusion of publications are available in Table S2.

### 2.1. Search Strategy

A systematic search of the literature in the Scopus and PubMed databases was performed (15 September 2022), using the following terms: anticoagulation monitoring; ECMO, extracorporeal membrane oxygenation, ECLS, ELS; and adverse events, complications, death, mortality (Table S3). To ensure the completeness of the search, we assessed the gray literature and the references of the included articles. All studies reporting on (a) ECMO support, (b) adverse events, (c) anticoagulation monitoring using aPTT, and (d) with more than 50 patients were included. Exclusion criteria were: (a) publications considering fewer than 50 patients, (b) selectively reporting on pediatric patients, and (c) duplicate publications. Furthermore, previous systematizations of evidence (reviews and meta-analyses), as well as articles reporting on data from the same center, or from the ELSO registry, were excluded to avoid potential overlap of patients. Finally, all publications with a main focus other than anticoagulation during ECMO support or reported in languages other than English were excluded. Following the practice used in prior studies, we chose a study sample size of 50, to exclude the potential impact of small studies and case reports. A detailed description of all study restrictions is provided in Table S2.

The articles screening was conducted by two independent authors (DJ, SR). Potential conflicts in publications selection were solved by a consensus within a team. 

### 2.2. Data Extraction and Synthesis

Two authors (DJ, SR) independently extracted the basic information from every study, including patient demographics, reported anticoagulation strategy and monitoring, complications, mortality, and technical information regarding ECMO support. The definitions of the reported outcomes were those adopted by the authors of the selected articles. A summary of the extraction process and data is provided in Table S4.

Finally, we performed simple calculations to standardize the results of included articles: (a) outcomes reported as a percentage were computed into numerical values, (b) if the outcome was reported only for certain groups, the overall sum was computed, and (c) median values were transformed into means with standard deviations. All calculations were performed by two authors independently (SR, CO).

### 2.3. Quality Assessment

The methodological quality of the included articles was evaluated with the Newcastle–Ottawa scale (NOS) [21]. Studies scored with at least 7 NOS stars were considered to be of good quality and with at least 5 as fair, whereas all articles with fewer than 5 stars were classed as low-quality (see Table 1). The quality of publications was assessed by two authors independently (CO, SR); any disagreement was resolved through a team consensus.

### 2.4. Statistical Assessment

Statistical analyses and visualizations of results were performed using the R software environment version 4.2.2 (“meta” and “metafor” packages; R Core Team 2022: R: A language and environment for statistical computing. R Foundation for Statistical Computing, Vienna, Austria). The comparison of patients with and without thromboembolic events was performed using the standardized mean differences (SMD). The SMDs were estimated by pooling the individual results of the study, using random or fixed effects models. The inverse variance methods with logit transformation were used for the pooled estimate of single proportions. Heterogeneity was explored by τ^2^ statistics and Cochran’s Q test, quantified with the I^2^ statistic. We assessed the publication bias using the Egger’s test and funnel plot. A significance level of 0.05 was employed.

## 3. Results

### 3.1. Search Results and Included Studies

**Figure 1:**
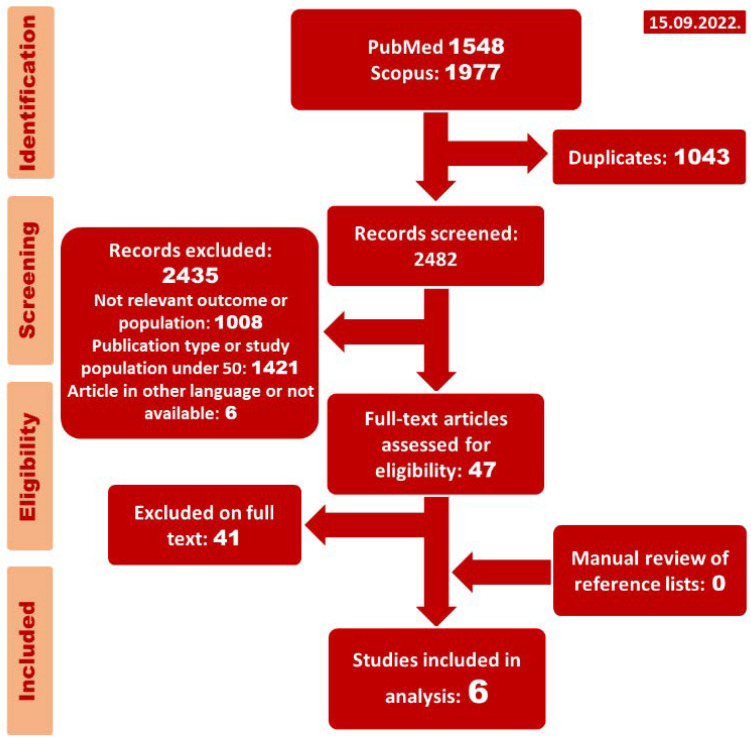
Preferred Reporting Items for Systematic Reviews and Meta-analyses (PRISMA) flowchart.

Our literature search yielded 3525 publications, 1977 in Scopus and 1548 in the PubMed database (up to 15 September 2022). In the next step, we excluded 2435 publications, 1421 due to the type of publication and 1008 for addressing irrelevant outcomes; see Figure 1. The main excluded publications are provided in Table S5. Thereby, 47 publications were selected for full-text assessment, of which 41 were further disqualified. Finally, we included six studies, with five reporting on the aPTT values and therefore being included in the meta-analysis.

The main characteristics of the included studies are reported in Table 1. The analyzed studies reflect the situation in France (*n* = 3), the USA (*n* = 2), and Germany (*n* = 1). Authors of the included studies obtained the outcome data through diverse prospective or retrospective databases, registers, and hospital records. The quality assessment identified four works of good quality, and two of fair quality; see Table 1.

### 3.2. Patient Population and Outcomes

Between 2006 and 2019, 1728 patients received V-A ECMO support, with 1463 being included in the meta-analysis. The included patient population was, on average, 57 ± 14 years old, and 69% were male. The main extracorporeal life support indications were postcardiotomy shock, acute cardiac failure, acute coronary syndrome, and cardiac arrest. Unfractionated heparin was used for anticoagulation, with an aPTT monitoring goal set between 45 and 80 s; see Table 1.

Bleeding (pooled rate 43%; 95% CI 28.4; 59.5) and any kind of thrombosis (pooled 36%; 95% CI 21.7; 53.7) were the complications most often reported. Within thromboembolic events, the brain and ECMO circuit were the most frequent thrombosis sites; see Table 2 and Table S6. In total, 765 of 1471 (pooled: 54%) patients did not survive to discharge. Definitions of thromboembolic events, as adopted by the authors, are provided in Table S7.

Finally, with regard to the ECMO technical data, the use of a centrifugal pump was reported in three studies, and none of the studies provided information on the circuit coating. The anticoagulation included UFH in all six studies, with two studies reporting the additional use of an alternative drug (due to heparin-induced thrombocytopenia—HIT 2 suspicion); see Table S7. Continuous anticoagulation use was reported in all publications.

### 3.3. Thromboembolic Events and aPTT

The majority of studies aimed to investigate the incidence of adverse events and to identify potential risk factors for thromboembolic and bleeding events in patients receiving V-A ECMO support; see Table 3. Three studies reported on ischemic stroke, two on mixed, and one on cannula-associated deep-vein thrombosis. None of the authors found any association of aPTT values with the occurrence of thromboembolic events; see Table 3.

**Figure 2:**
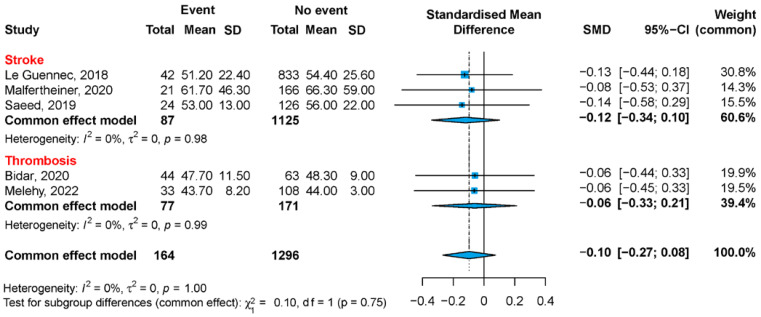
Forest plot: Average aPTT values among V-A ECMO patients. V-A ECMO = veno-arterial extracorporeal membrane oxygenation; aPTT = activated partial thromboplastin time [22,23,24,25,27].

**Figure 3:**
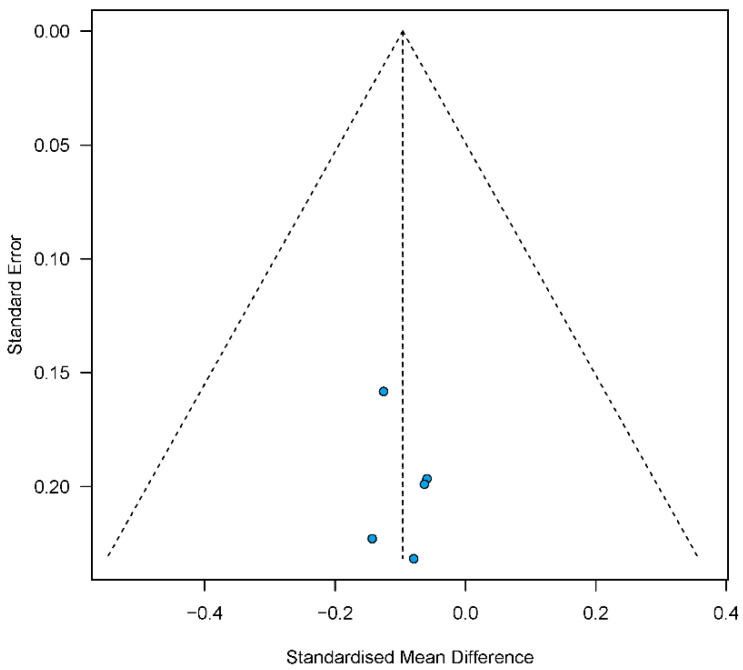
Funnel plot of publication bias.

Five studies reported average aPTT values, three for ischemic stroke and two for any thrombosis (Figure 2 and Figure 3). There was no difference in the reported aPTT values of patients who had any kind of thromboembolic event, compared to those without (SMD −0.10; 95% CI −0.27; 0.08, *p* = 0.273), with a heterogeneity of I^2^ = 0% (95% CI 0.0; 79.2, Q = 0.2, Tau^2^ = 0, *p* = 0.997). Within the subgroups (ischemic stroke or any thrombosis), the pooled SMD was slightly different in studies reporting on ischemic stroke (SMD −0.12; 95% CI −0.34; 0.10 vs. SMD −0.10; 95% CI −0.27; 0.08). Moreover, we calculated an average aPTT of 53 ± 26 s in publications reporting on ischemic stroke presence (56 ± 32 s without event), and 45 ± 12 s in publications focusing on any thrombosis (46 ± 6 s without event). Finally, there was no difference in age in the groups compared (five studies, *n* = 1460, *p* = 0.963); see Figure S1.

### 3.4. Thrombosis and Mortality

The mortality of ECMO patients was not the main focus of included studies; however, four authors provided information on in-hospital mortality, with a pooled rate of 54%. Five studies (*n* = 1463) provided data on both thromboembolic events and mortality, with a pooled in-hospital mortality of 53% (95% CI 31.0; 74.2) for patients with, and 32% (95% CI 20.0; 46.5) for patients without, thromboembolic events; see Table S6. Studies reporting on stroke showed higher in-hospital mortality (67%, 95% CI 55.5; 75.9) compared to studies reporting on any thrombosis (47%, 95% CI 43.5; 49.8).

## 4. Discussion

The present literature review and meta-analysis evaluated the association of aPTT-guided anticoagulation monitoring and thrombosis in patients receiving V-A ECMO support. The use of aPTT for UFH monitoring is seen as the standard of care, aiming to avoid extracorporeal circuit clotting by setting a certain aPTT anticoagulation goal. Analyzing six studies comprising 1728 patients, we did not find any difference in reported aPTT values for patients with and without thrombotic events. Finally, within the qualitative analysis, none of the included studies observed an association of aPTT with thrombosis.

### 4.1. ECMO Support and aPTT

Despite a few upcoming randomized controlled trials (SAFE-ECMO NCT04997265; RATE NCT04536272; and A-FREE ECMO NCT04273607), current praxis in anticoagulation monitoring is based on weak evidence. Moreover, previous research focused on the comparison of different anticoagulation agents and outcomes, rather than on analyzing anticoagulation monitoring itself. The ELSO recommends using a systemic anticoagulation to prevent circuit clotting, with a goal-aPTT of 1.5–2.5 times the patient’s baseline (40–80 s) [13]. However, worldwide consensus is still missing, and the optimal anticoagulation strategy remains unclear [28,29,30]. In the included studies, we observed diverse aPTT goals, ranging from 45 to 80 s. Interestingly, although the mean aPTT values were within the targeted range, thrombotic and/or bleeding events occurred to a significant extent.

Evidence if the recommended targets for aPTT-guided anticoagulation are too high is missing, although hemorrhage has been proven to be a significant risk factor of poor patient outcome and mortality during ECMO support [5,6,31,32,33]. Comparing the adverse events profile or thromboembolic complications only in a recent systemic literature review, the outcome of the patients receiving systemic anticoagulation did not differ from those with an “anticoagulation-free” approach [12]. However, an “anticoagulation-free” regimen must be critically questioned in this context, as some of the patients analyzed still received some kind of anticoagulation therapy.

Systematized evidence of the association of aPTT levels with the occurrence of thrombosis or bleeding in ECMO patients is missing, and time-guided anticoagulation monitoring for UFH has certain limitations. Effective therapeutic drug monitoring requires a linear relationship between the dose and clinical effect, but heparin has been shown to have high pharmacokinetic variability [34,35,36]. This might be even more pronounced in severely ill patients with their varying antithrombin levels and kallikrein-kinin pathway or factor XII deficiencies, resulting in spontaneous prolongation of aPTT [37,38,39]. On the other hand, the sensitivity of aPTT reagents to UFH varies, possibly resulting in different aPTT results.

In this context, the use of anti-factor-Xa for UFH monitoring in intensive care is becoming increasingly important, but as the data are still controversial, further studies are needed before it can be implemented in the clinical routine [10,14,30,34,40,41,42,43,44]. However, due to its general availability and low cost, aPTT continues to be a widely available monitoring method and is often the only available alternative at some facilities. Ongoing clinical studies should provide new evidence on reduced anticoagulation management during ECMO support to help tailor future recommendations.

### 4.2. Thromboembolic Events in Patients Receiving ECMO Support

The existing literature comprises mostly retrospective works with small patient populations. Recommendations on reporting or a standardized definition of thrombosis in patients with ECMO support is missing, leading to inconsistent documentation of adverse events. Therefore, we provide the definitions used by the authors of the studies analyzed, including information on the thrombosis incidence from selected studies, with a focus on thromboembolic events. As more conformity means more accuracy, it can be assumed that our data on thrombosis are more consistent with reality.

We obtained a pooled rate of 36% for thrombosis, which is significantly higher than in the literature [9,45,46,47]. This may be attributed to the larger sample size, but also to a potential selection bias related to our search strategy. We focused on, and finally included only, works providing data on both aPTT monitoring and thrombosis. Moreover, the authors of the included studies aimed to identify the risk factors for thrombosis and/or bleeding, which could lead to more precise identification of adverse events, reducing the rate of potentially missed thromboembolic events. The problem of the rather low reported thrombosis incidence is often referred to in the literature, but high-quality prospective or randomized clinical trials do not exist. The low incidence of thromboembolic events could be explained by high underestimation in retrospective studies [9,48]. Furthermore, the lack of regular radiological investigations and post-mortem examinations, or missing data on these diagnostic procedures in retrospective databanks, may lead to a false low rate of thrombosis.

Ischemic stroke occurred in 11% of patients, ranging from 5% to 17% in line with a recent meta-analysis [9]. However, the analysis of ELSO registry patients reported an ischemic stroke incidence of 4%, without significant changes in the last 25 years (3.6% in 1992–2013, and 3.9% in 2013–2017) [49,50]. The authors note that the true prevalence is likely higher than reported, due to potential underestimation and the retrospective nature of the register. Moreover, the in-hospital mortality of patients with ischemic stroke was lower compared to those with intracranial hemorrhage (76% vs. 86%), but central nervous system complications are still associated with significant morbidity, poor outcome, and high mortality [50].

Despite the significant development of extracorporeal life technology, the rate of ECMO circuit and membrane clotting is still high. Based on the data from three studies, we found ECMO circuit-related thrombosis in 11% of patients, slightly higher than in the literature [9]. Technical ECMO characteristics and complications are still underreported, and reporting on complications remains limited by the high heterogeneity of the data provided. Moreover, two studies reported on the use of argatroban or bivalirudin in case of HIT 2 [25,27]. Evidence on the use of alternative anticoagulants is increasing, and future studies could lead to new recommendations [51,52,53,54,55].

Finally, hemorrhage was observed in almost one-half of patients, similar to the rate reported in the literature [9,45,46,56]. We calculated a pooled in-hospital mortality of 54%, corresponding to the latest ELSO International Summary of Statistics report (55%, based on 43331 V-A ECMO runs in adults) and meta-analysis [9,57]. Furthermore, a qualitative analysis of data on both thromboembolic events and mortality revealed no difference in mortality between patients suffering thrombosis or not in three studies [22,23,24], while two did not provide information on mortality and, in one, the risk of death was higher for patients who suffered a nonhemorrhagic stroke [27].

### 4.3. Future Developments and Outlook

Future research on potential predictors and risk factors for thromboembolic events, anticoagulation strategies and monitoring, patient-related factors, ECMO systems, and surgical techniques is warranted. The severity of adverse events, especially in the case of bleeding complications and the potential for permanent injury or death, led to ongoing research of other anticoagulation possibilities or even anticoagulation-free ECMOs. Emerging evidence on higher morbidity and mortality related to hemorrhage rather than thromboembolic complications resulted in reports on the feasibility of ECMO support without systemic anticoagulation [12,58,59,60]. However, these results should be interpreted cautiously, as some type of anticoagulation was used in these studies.

The information on anticoagulation monitoring is still limited and prospective studies are missing. Despite extensive research, we are still not able to evaluate the global function of coagulation in vivo or to continuously monitor it. The evidence on more appropriate methods of UFH monitoring is growing, and current studies promote the use of anti-Xa assays or viscoelastic methods [7,10,17,61,62,63,64].

Finally, potentially modifiable risk factors for thromboembolic events should be recognized early to guarantee appropriate management. Due to the substantial heterogeneity in reporting, systematization of adverse events is complex. None of the authors described an association of aPTT-guided monitoring or any aPTT threshold as risk factor for thromboembolic events. Within other potentially associated factors, two authors identified central cannulation as a risk factor for thromboembolic events. Furthermore, ECMO duration, concurrent infection, and surgical venting were associated with thrombosis. Platelets above 350 giga/L, cardiac surgery, autoimmune disease, age, lactate, delta D dimer, and hemolysis were identified as potential risk factors for ischemic stroke. In one study, preexisting anticoagulation was associated with thrombosis [25], and in another it was considered a protective factor for cannula-associated deep-vein thrombosis [22]; see Table 3. These findings warrant further research.

### 4.4. Strengths and Limitations

The strengths of our work include the international sample of studies comprising 1728 V-A ECMO patients, with at least fair methodological quality. Moreover, we excluded works from the ELSO registry and those from the same centers to control for potential patient overlap. Despite all the benefits international databases may bring, these registers could be limited in representing the real-life situation globally as the institutions participating in the ELSO registry are not a random sample, but centers with ELSO membership. Therefore, selection and notification bias may exist. Given the above, we present systematized evidence based on a search of large scientific databases, independent of center status. Finally, our work is reported in accordance with the PRISMA guidelines; see Table S1.

However, our study has certain limitations. The quality of the evidence provided is as strong as all included publications, given their single-center and retrospective nature. However, we cannot exclude retrieval or publication bias. To reduce the potential effect of small studies, we excluded all studies with fewer than 50 V-A ECMO patients. Most of the authors reported on adverse events, but omitted to give a precise or standardized definition of them. This made further comparisons complicated.

The comparison of reported aPTT values between studies may be difficult due to the variety of methods and assays. However, this should have a rather small effect due to minimal alterations, and we collected information on the final study conclusions regarding the potential association of aPTT and thromboembolic events. This conclusion is based on evaluation of the whole study data. As recommendations on ECMO-related outcome reporting are still not available, the variety of research questions led to diverse reporting of outcomes. This resulted in a systematization of the evidence into mixed thrombotic events and ischemic stroke. Finally, we cannot clarify if the reported complications are a consequence of V-A ECMO itself or the underlying illness independent of support.

Heterogeneity is a recognized limitation of observational retrospective works, which may imply the increased variance of analyzed publications. Thus, some results should be interpreted cautiously, as the observed heterogeneity may restrict their meta-analytic portion.

Finally, our robust inclusion and clear exclusion criteria resulted in the inclusion of six studies. The available evidence on aPTT-guided anticoagulation monitoring and thrombosis occurrence is still scarce and predominated by reports on bleeding complications. However, we were able to systematize the evidence and perform a meta-analysis.

## 5. Conclusions

Despite the tremendous development of critical care, aPTT-guided systemic anticoagulation is still the standard monitoring tool in patients receiving ECMO support. The existing aPTT anticoagulation goals are based on weak evidence and have never been validated in ECMO patients. We did not find any association of aPTT values with thrombosis occurrence. Further evidence and new randomized trials are needed to clarify the true incidence of thromboembolic events and the best anticoagulation and monitoring strategy in V-A ECMO patients. Until these data are available, our work is limited, and showed no difference in aPTT values of patients with and without thromboembolic events.

## Figures and Tables

**Table 1:** Characteristics of the included studies (*n* = 1728).

Author	Country, Study Period	Prospective Study	Sample Size	Age (Years)	ECMO Duration (Days)	Goal aPTT	Population	Comparison	NOS
Bidar et al. [22]	France, 2018–2019	No	107	54	8	1.5–2x normal	Acute on chronic cardiac failure, postcardiotomy, cardiac arrest, and acute coronary syndrome	Presence of cannula-associated deep-vein thrombosis	8
Le Guennec et al. [23]	France, 2006–2014	No	878	-	-	1.5–2x baseline	Postcardiotomy, cardiac arrest, AMI, chronic dilated cardiomyopathy, and other	Presence of ischemic stroke	7
Malfertheiner et al. [24]	Germany, 2011–2016	No	187	58.5	4	50–80	ECPR, LCOS, and postcardiotomy	Presence of intracranial ischemia	6
Melehy et al. [25]	USA, 2007–2019	No	141	65	5.2	45–60, and 60–80	Postcardiotomy shock	Presence of thrombosis	7
Moussa et al. [26]	France, 2015–2019	No	265	55 ^a^	-	-	LCOS, primary graft dysfunction, and myocardial infarction	Association of anti-Xa and aPTT with thrombosis	5
Saed et al. [27]	USA, 2012–2017	No	150	55 ^a^	-	50–70	Postcardiotomy and AMI	Presence of nonhemorrhagic stroke	7

^a^ Mean value; NOS = Newcastle–Ottawa scale score; aPTT = activated partial thromboplastin time; AMI = acute myocardial infarction; VA-ECMO = veno-arterial extracorporeal membrane oxygenation; ECPR = extracorporeal cardiopulmonary reanimation; LCOS = low cardiac output syndrome.

**Table 2:** Reporting of ECMO-related outcomes and adverse events rate.

Outcome		Number of Studies Reporting Data (Events)	Pooled Rate (95% CI)	*I^2^%* (*p*-Value)	Reported Range (%)
**Thrombosis**					
	Any thrombosis	3 (179)	36.2 (21.7; 53.7)	93 (<0.001)	23.4–55.1
	Ischemic stroke	4 (133)	11.2 (5.7; 20.7)	94 (<0.001)	4.8–17.4
	Limb ischemia	3 (29)	5.7 (4.0; 8.1)	0 (0.868)	5.0–6.5
	ECMO circuit and membrane clot	3 (52)	10.6 (8.2; 13.7)	66 (0.054)	6.4–15.9
	HIT	2 (8)	4.0 (2.0; 7.8)	60 (0.112)	0.9–5.0
**Hemorrhage**					
	Any bleeding	3 (244)	43.3 (28.4; 59.5)	92 (<0.001)	28.0–56.6
	Cerebral hemorrhage	4 (39)	2.8 (1.6; 5.1)	64 (0.041)	1.4–5.9
	Cannulation site bleeding	7 (184)	14.0 (10.7; 18.1)	71 (0.002)	7.1–17.4
**In-hospital mortality**		4 (765)	54.4 (47.6; 61.0)	81 (0.001)	49.2–65.2

ECMO = extracorporeal membrane oxygenation; HIT = heparin-induced thrombocytopenia.

**Table 3:** Main aims and outcomes of the included studies (*n* = 1728).

Author	Main Study Aim	Thrombotic Event	Association of Monitoring with Thrombosis	Other Factors Associated with Thrombosis/Author Comments
Bidar et al. [22]	Incidence and risk factors for thrombosis in VA-ECMO patients after decannulation	cannula-associated deep-vein thrombosis	No: The median aPTT ratio and percentage of days with aPTT in the therapeutic range did not differ between groups.	Multivariable model identified ECMO duration and concurrent infection as independent risk factors for cannula-associated deep-vein thrombosis. Older age and prior anticoagulation were protective factors.
Le Guennec et al. [23]	Frequency and risk factors for structural brain injury	Ischemic stroke	No: No relevant association could be established between aPTT and ischemic stroke.	Platelets above 350 giga/L (OR 3.8, 95% CI 1.4–10.7) at ECMO start were associated with stroke. Ischemic stroke does not seem to be associated with higher mortality.
Malfertheiner et al. [24]	Incidence of ischemia	Ischemic stroke	No: Atrial fibrillation, hypocapnia, lower aPTT, INR values or lower heparin doses at any time during ECMO support were not associated with the incidence of stroke.	Cardiac surgery prior to ECMO (OR 2.8, 95% CI 1.1–7.1), autoimmune disease (OR 4.2, 95% CI 1.2–15.0), age (OR 2.5, 95% CI 1.5–4.1), lactate (OR 1.1, 95% CI 1.0–1.2), delta D dimer (OR 1.4, 1.1–1.8), and cannulation of ascendence aorta were associated with stroke (OR 4.0, 95% CI 1.1–14.3).
Melehy et al. [25]	Impact of anticoagulation thrombosis	Thrombosis	No: aPTT before event was not associated with thrombosis.	Predictors of thrombosis included anticoagulation before event (OR 0.4, 95% CI 0.2–0.8), surgical venting (OR 3.1, 95% CI 1.3–7.3), and central cannulation (OR 2.1, 95% CI 1.0–4.1) before event.
Moussa et al. [26]	Association of anti-factor Xa and aPTT with thrombosis	Thrombosis	No: Daily maximum, minimum and mean anti-factor Xa and aPTT values were not associated with thrombosis.	The values of anti-factor Xa and aPTT were highly discordant. These results should favor the use of anti-factor Xa.
Saeed et al. [27]	Identification of risk factors for nonhemorrhagic stroke	Ischemic stroke	No: At the time of the stroke, aPTT was in the therapeutic range.	Hemolysis at low levels is associated with nonhemorrhagic stroke (aHR 7.6, 95% CI 2.2–25.9).

ECMO = extracorporeal membrane oxygenation; aPTT = activated partial thromboplastin time; va-ECMO = veno-arterial extracorporeal membrane oxygenation; HR = hazard ratio; OR = odds ratio; CI = confidence intervals.

## Data Availability

The datasets used and analyzed during the current study can be made available by the corresponding author on reasonable request.

## Supplementary Materials

The following supporting information can be downloaded at: https://www.mdpi.com/article/10.3390/jcm12093224/s1, Table S1. Preferred Reporting Items for Systematic review and Meta-Analysis (PRISMA) 2020 checklist; Table S2. PICOS criteria for inclusion and exclusion of publications; Table S3. Search strategy; Table S4. Data extraction and synthesis protocol; Table S5. Main excluded studies; Table S6. Reported adverse events in the included studies; Table S7. Patient anticoagulation, definition of event and ECMO characteristics of the included studies; Figure S1. Forest plot: Average age of patients requiring extracorporeal membrane oxygenation support.
