# Supplementary material for: Association of aPTT-Guided Anticoagulation Monitoring with Thromboembolic Events in Patients Receiving V-A ECMO Support: A Systematic Review and Meta-Analysis
Source: J Clin Med. 2023 Apr 30;12(9):3224. doi: 10.3390/jcm12093224 (PMC10179156; doi:10.3390/jcm12093224)
Supplement: Supplementary file 1 [file jcm-12-03224-s001.zip › jcm-2336395-supplementary.pdf]

Supplementary material

Association of aPTT-guided anticoagulation monitoring with thromboembolic events in patients receiving V-A ECMO support: A systematic review and meta-analysis

Contents

Table S1. Preferred Reporting Items for Systematic review and Meta-Analysis (PRISMA) 2020 checklist

Table S2. PICOS criteria for inclusion and exclusion of publications

Table S3. Search strategy

Table S4. Data extraction and synthesis protocol

Table S5. Main excluded studies

Table S6. Reported adverse events in the included studies

Table S7. Patient anticoagulation, definition of event and ECMO characteristics of the included studies

Figure S1. Forest plot: Average age of patients requiring extracorporeal membrane oxygenation support

Table S1. Preferred Reporting Items for Systematic review and Meta-Analysis (PRISMA) 2020 checklist

Section and Topic	Item #	Checklist item	Location where item is reported
**TITLE**			
Title	1	Identify the report as a systematic review.	Page 1
**ABSTRACT**			
Abstract	2	See the PRISMA 2020 for Abstracts checklist.	Page 1
**INTRODUCTION**			
Rationale	3	Describe the rationale for the review in the context of existing knowledge.	Page 2
Objectives	4	Provide an explicit statement of the objective(s) or question(s) the review addresses.	Page 2-3
**METHODS**			
Eligibility criteria	5	Specify the inclusion and exclusion criteria for the review and how studies were grouped for the syntheses.	Page 2-3
Information sources	6	Specify all databases, registers, websites, organisations, reference lists and other sources searched or consulted to identify studies. Specify the date when each source was last searched or consulted.	Page 2-3
Search strategy	7	Present the full search strategies for all databases, registers and websites, including any filters and limits used.	Page 2-3, Supplementary
Selection process	8	Specify the methods used to decide whether a study met the inclusion criteria of the review, including how many reviewers screened each record and each report retrieved, whether they worked independently, and if applicable, details of automation tools used in the process.	Page 2-3
Data collection process	9	Specify the methods used to collect data from reports, including how many reviewers collected data from each report, whether they worked independently, any processes for obtaining or confirming data from study investigators, and if applicable, details of automation tools used in the process.	Page 2-3
Data items	10a	List and define all outcomes for which data were sought. Specify whether all results that were compatible with each outcome domain in each study were sought (e.g. for all measures, time points, analyses), and if not, the methods used to decide which results to collect.	Page 2-3, Supplementary
	10b	List and define all other variables for which data were sought (e.g. participant and intervention characteristics, funding sources). Describe any assumptions made about any missing or unclear information.	Page 2-3, Supplementary
Study risk of	11	Specify the methods used to assess risk of bias in the included studies, including details of the tool(s) used, how many	Page 2-3

Section and Topic	Item #	Checklist item	Location where item is reported
bias assessment		reviewers assessed each study and whether they worked independently, and if applicable, details of automation tools used in the process.	
Effect measures	12	Specify for each outcome the effect measure(s) (e.g. risk ratio, mean difference) used in the synthesis or presentation of results.	Page 2-3
Synthesis methods	13a	Describe the processes used to decide which studies were eligible for each synthesis (e.g. tabulating the study intervention characteristics and comparing against the planned groups for each synthesis (item #5)).	Page 2-3
	13b	Describe any methods required to prepare the data for presentation or synthesis, such as handling of missing summary statistics, or data conversions.	Page 2-3
	13c	Describe any methods used to tabulate or visually display results of individual studies and syntheses.	Page 2-3, Supplementary
	13d	Describe any methods used to synthesize results and provide a rationale for the choice(s). If meta-analysis was performed, describe the model(s), method(s) to identify the presence and extent of statistical heterogeneity, and software package(s) used.	Page 2-3
	13e	Describe any methods used to explore possible causes of heterogeneity among study results (e.g. subgroup analysis, meta-regression).	Page 3
	13f	Describe any sensitivity analyses conducted to assess robustness of the synthesized results.	Page 3
Reporting bias assessment	14	Describe any methods used to assess risk of bias due to missing results in a synthesis (arising from reporting biases).	Page 3
Certainty assessment	15	Describe any methods used to assess certainty (or confidence) in the body of evidence for an outcome.	Page 3
**RESULTS**			
Study selection	16a	Describe the results of the search and selection process, from the number of records identified in the search to the number of studies included in the review, ideally using a flow diagram.	Page 3-8
	16b	Cite studies that might appear to meet the inclusion criteria, but which were excluded, and explain why they were excluded.	Supplementary
Study characteristics	17	Cite each included study and present its characteristics.	Page 3-8
Risk of bias in studies	18	Present assessments of risk of bias for each included study.	Page 1
Results of	19	For all outcomes, present, for each study: (a) summary statistics for each group (where appropriate) and (b) an effect estimate	Page 3-8,

Section and Topic	Item #	Checklist item	Location where item is reported
individual studies		and its precision (e.g. confidence/credible interval), ideally using structured tables or plots.	Supplementary
Results of syntheses	20a	For each synthesis, briefly summarise the characteristics and risk of bias among contributing studies.	Page 3-8, Supplementary
	20b	Present results of all statistical syntheses conducted. If meta-analysis was done, present for each the summary estimate and its precision (e.g. confidence/credible interval) and measures of statistical heterogeneity. If comparing groups, describe the direction of the effect.	Page 3-8, Supplementary
	20c	Present results of all investigations of possible causes of heterogeneity among study results.	Page 3-8 Supplementary
	20d	Present results of all sensitivity analyses conducted to assess the robustness of the synthesized results.	Supplementary
Reporting biases	21	Present assessments of risk of bias due to missing results (arising from reporting biases) for each synthesis assessed.	Page 3-8
Certainty of evidence	22	Present assessments of certainty (or confidence) in the body of evidence for each outcome assessed.	Page 3-8
**DISCUSSION**			
Discussion	23a	Provide a general interpretation of the results in the context of other evidence.	Page 9-12
	23b	Discuss any limitations of the evidence included in the review.	Page 9-12
	23c	Discuss any limitations of the review processes used.	Page 9-12
	23d	Discuss implications of the results for practice, policy, and future research.	Page 10-12
**OTHER INFORMATION**			
Registration and protocol	24a	Provide registration information for the review, including register name and registration number, or state that the review was not registered.	Page 2-3
	24b	Indicate where the review protocol can be accessed, or state that a protocol was not prepared.	Page 2-3
	24c	Describe and explain any amendments to information provided at registration or in the protocol.	Page 2-3
Support	25	Describe sources of financial or non-financial support for the review, and the role of the funders or sponsors in the review.	Page 12
Competing interests	26	Declare any competing interests of review authors.	Page 12
Availability of	27	Report which of the following are publicly available and where they can be found: template data collection forms; data	Page 12

Section and Topic	Item #	Checklist item	Location where item is reported
data, code and other materials		extracted from included studies; data used for all analyses; analytic code; any other materials used in the review.	

From: Page MJ, McKenzie JE, Bossuyt PM, Boutron I, Hoffmann TC, Mulrow CD, et al. The PRISMA 2020 statement: an updated guideline for reporting systematic reviews. BMJ 2021;372:n71. doi: 10.1136/bmj.n71.

Table S2. PICOS criteria for inclusion and exclusion of publications

**Parameter**	**Inclusion criteria**	**Exclusion criteria**
Population	- >13 years	- <13 years
Intervention	- ECMO support<br>- UFH Anticoagulation<br>- aPTT-guided anticoagulation monitoring	- ECMO patients transport,<br>- Multiple runs,<br>- Patient overlapping (i.e., multiple studies with the same patient population, ELSO registry studies - overlap with submitting center)<br>- Studies not reporting on aPTT-guided anticoagulation monitoring<br>- Anticoagulation free ECMO
Comparator	None	None
Outcomes	Primary:<br>- aPTT-guided anticoagulation monitoring<br>- Thromboembolic events<br>Secondary:<br>- Clinical and demographic characteristics of ECMO support patients,<br>- Reported complications,<br>- Mortality (in-hospital).	-
Study design	- Study population of 50 or more patients,<br>- Randomized trials,<br>- Prospective and retrospective cohort studies,<br>- Cross-sectional studies<br>- Grey literature	- Study population of 49 or less patients,<br>- Case reports,<br>- Systematic reviews,<br>- Meta-analyses,<br>- Conference abstracts,<br>- Short reports,<br>- Letters

PICOS patients/population, intervention, comparator, outcomes, study design, *ELSO* Extracorporeal Life Support Organization, *ECMO* extracorporeal membrane oxygenation, *UFH* unfractionated heparin, *aPTT* activated prothrombin time

Table S3. Search strategy

**Database: PubMed**
Date searched: 15.09.2022.<br>Records retrieved: 1548<br>Used terms:<br>Extracorporeal membrane oxygenation[MeSH Terms]; ECMO; extracorporeal life support; ECLS<br>"Death"[Mesh]; complications; adverse; "Embolism and Thrombosis"[Mesh]; "Stroke"[Mesh]; "Hemorrhage"[Mesh];<br>"Blood Coagulation Tests"[Mesh]; anticoagulation; anticoagulation monitoring; "Drug Monitoring"[Mesh]; monitoring; aptt[Title/Abstract]; "partial thromboplastin time"[MeSH Terms]; anti-factor Xa; anti Xa; Activated clotting time; ACT; "Thrombelastography"[Mesh]; viscoelastic testing.<br>Filters: Humans
**Database: Scopus**
Date searched: 15.09.2022.<br>Records retrieved: 1977<br>Used terms:<br>"Blood Coagulation Tests"; "anticoagulation monitoring"; anticoagulation; "Drug Monitoring"; monitoring; "partial thromboplastin time"; apt; ACT; "Activated clotting time"; "anti Xa"; "anti-factor Xa"; "viscoelastic testing"; thrombelastography;<br>Death; complication*; adverse; thrombosis; stroke; hemorrhage; bleeding;<br>"extracorporeal life support"; ECLS "extracorporeal membrane oxygenation"; ECMO.<br>LIMIT-TO "Human"

Table S4. Data extraction and synthesis protocol

**Characteristics**	**Description**
Study	First author, country and institution name, year of publication, information on used register, study design, follow-up and recruitment period, ECMO support indications, study sample, and compared groups.
Clinical and demographic	Sex, age, cardiac arrest before ECMO, body mass index, the sequential organ failure assessment (SOFA) score, simplified acute physiology score (SAPS II and III) score, acute physiology and chronic health evaluation II (APACHE II) score, duration of ECMO support, and type of cannulation.
Complications	Total number of thrombotic events, venous thrombosis, arterial thrombosis, deep vein thrombosis, ischemic stroke, pulmonary embolism, cannula associated thrombosis, limb ischemia, ECMO circuit and membrane clot; major bleeding events (ELSO), hemorrhagic events, cannulation and surgical site bleeding, pulmonary bleeding, pericardial bleeding, gastrointestinal tract hemorrhage, intracerebral hemorrhage; renal replacement therapy; acute kidney injury, and sepsis.
Mortality	Mortality during intensive care unit stay, and in-hospital mortality.
Anticoagulation regime	Type of anticoagulation used, information on monitoring, use of continuous infusion, the range of the goal anticoagulation (aPTT in seconds), the use of unfractionated heparin (UFH) coating, and the authors conclusion on potential association of anticoagulation monitoring with thromboembolic events.
ECMO-related technical characteristics	ECMO pump type, machine used, and equipment-related adverse events.

APACHE II acute physiology and chronic health evaluation II, *aPTT* activated partial thromboplastin time, *ELSO* extracorporeal life support organization, *ECMO* extracorporeal membrane oxygenation, *SOFA* sequential organ failure assessment, *UFH* unfractionated heparin, *SAPS* simplified acute physiology score

Table S5. Main excluded studies

Study (author and year)	Number of patients	Reason for exclusion
Treml et al. [1]	358	No outcome of interest
Yie et al. [2]	60	No outcome of interest
Weber et al. [3]	281	No outcome of interest
Fitousis et al. [4]	122	No outcome of interest
Wilcox et al. [5]	52	No outcome of interest
Raman et al. [6]	52	No outcome of interest
Wood et al. [7]	203	No outcome of interest
Marinoni et al. [8]	53	No outcome of interest
Lim et al. [9]	320	Missing distinction between thrombotic and hemorrhagic complications
Park et al. [10]	93	Missing report on anticoagulation monitoring
Laimoud et al. [11]	65	Risk of patient overlap
Melehy et al. [12]	90	Risk of patient overlap
Cooper et al. [13]	103	Publication type
Brunet et al. [14]	64	No outcome of interest
Ruggeri et al. [15]	188	No outcome of interest
Becher et al. [16]	10207	Risk of patient overlap

Table S6. Reported adverse events in the included studies

Adverse events	Author					
	**Bidar et al. [17]**	**Le Guennec et al. (23)**	**Malfertheiner et al. (25)**	**Melehy et al. (27)**	**Moussa et al. (28)**	**Saeed et al. [18]**
Bleeding	30	-	-	64	150	-
Surgical site bleeding	-	-	-	-	-	-
Cannulation site bleeding	-	-	-	10	46	-
Intracerebral hemorrhage	-	20	11	2	6	-
Ischemic stroke	-	42	21	-	46	24
Thrombosis	59	-	-	33	87	-
Venous thrombosis	44	-	-	-	-	-
Arterial thrombosis	15	-	-	-	-	-
Deep vein thrombosis	-	-	-	8	-	-
inferior vena cava	38	-	-	-	-	-
Extremity veins	7	-	-	-	-	-
Cannula associated thrombosis	-	-	-	-	-	-
Pulmonary embolism	-	-	-	-	-	-
Limb ischemia	7	-	-	7	15	-
Multiple thrombosis	4	-	-	-	-	-
HIT	1	-	-	7	-	-
Renal replacement therapy	-	-	-	60	-	-
Acute kidney injury	-	-	-	-	-	-
Sepsis	-	-	-	-	-	-
ECMO circuit and membrane clot	17	-	-	9	26	-
In-hospital mortality	-	432	122	75	136	-
In-hospital mortality (%)	-	49.4	65.2	53	51.3	-

ECMO Extracorporeal membrane oxygenation, *CNS* central nervous system, *HIT* Heparin-induced thrombocytopenia

Table S7. Patient anticoagulation, definition of event and ECMO characteristics of the included studies

Author	Anticoagulation (type)	Continuous UFH	UFH coating	Event definition	Pump type	Central / Periphery (%)
Bidar et al. [17]	UFH	Yes	-	Diagnosis of CaDVT was based on thrombus presence, absence of flow, and vein incompressibility (for peripheral veins), or thrombus presence (for inferior vena cava); PE was diagnosed using CT in case of clinically suspicion.	-	14/86
Le Guennec et al. [19]	UFH	Yes	-	Clinical signs suggestive of stroke (anisocoria, mydriasis, hemiplegia, asymmetry); brain injury was assessed using CT imaging. The haemorrhagic progression of ischemic stroke was classified as ischemic stroke.	-	15/85
Malfertheiner et al. [20]	UFH	Yes	-	Cerebral CT radiological reports were screened to identify evidence of cerebral pathology (including ischaemia and others). A secondary haemorrhagic transformation of ischemic stroke was considered as primary ischaemic.	Rotaflow or Cardiohelp device (Getinge), Deltastream (Medos) or Stöckert (Sorin Group)	10/90
Melehy et al. [21]	UFH and Argatroban[a]	Yes	-	Thrombotic events included deep vein thrombosis, limb ischemia, PE, ischemic stroke, intracardiac thrombosis, circuit thrombosis, and other arterial system thromboses. Echocardiography was used for identification of intracardiac thrombi. ECMO oxygenator was exchanged wenn a delta P was greater than 35mmHg, postoxygenator PO_2 less than 250mmHg, and based on the clinical assessment.	-	42/58
Moussa et al. [22]	UFH	Yes	-	The thrombosis included stroke, limb ischaemia and ECMO circuit changes due to thrombosis, and any thrombosis that consequently led to surgical or medical intervention or death.	Rotaflow (Getinge), Revolution (LivaNova Group) or Centrimag (Thoratec)	1/99
Saeed et al. [18]	UFH, Argatroban or Bivalirudin[a]	Yes	-	Non-hemorrhagic stroke included new neurological symptoms confirmed by neurologist and a cerebral CT presentation of acute cerebral infarction without the presence of hemorrhage.	Rotaflow (Getinge)	11/89

[a]Alternative anticoagulation used only for cases with heparin induced thrombocytopenia. *ECMO* extracorporeal membrane oxygenation, *ELSO* Extracorporeal Life Support Organization, *UFH* unfractionated heparin, *PE* pulmonary embolism, *CaDVT* cannula-associated deep-vein thrombosis, *CT* computed tomography

Figure S1. Forest plot: Average age of patients requiring extracorporeal membrane oxygenation support (stroke and thrombosis, p = 0.963).

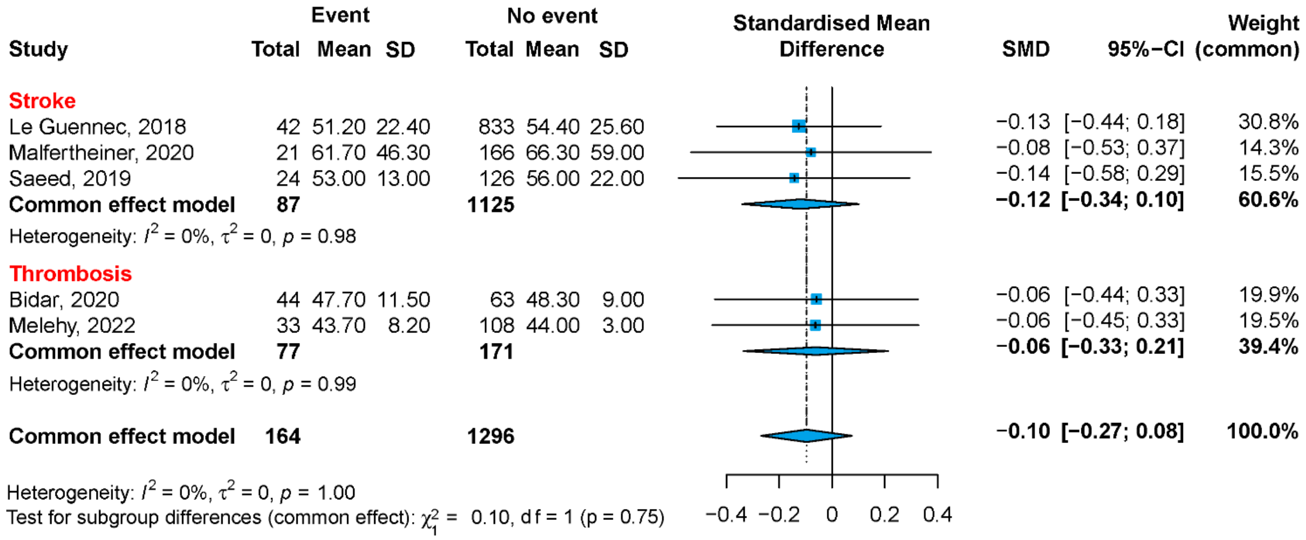

References

1. Treml, B.; Breitkopf, R.; Bukumirić, Z.; Bachler, M.; Boesch, J.; Rajsic, S. ECMO Predictors of Mortality: A 10-Year Referral Centre Experience. *Journal of clinical medicine* **2022**, *11*, doi:10.3390/jcm11051224.
2. Yie, K.; Chon, S.-H.D.; Na, C.-Y. Activated clotting time test alone is inadequate to optimize therapeutic heparin dosage adjustment during post-cardiopulmonary resuscitational extracorporeal membrane oxygenation (e-CPR). **2016**, *31*, 307-315, doi:10.1177/0267659115604710.
3. Weber, C.; Deppe, A.-C.; Sabashnikov, A.; Slottosch, I.; Kuhn, E.; Eghbalzadeh, K.; Scherner, M.; Choi, Y.-H.; Madershahian, N.; Wahlers, T. Left ventricular thrombus formation in patients undergoing femoral veno-arterial extracorporeal membrane oxygenation. **2018**, *33*, 283-288, doi:10.1177/0267659117745369.
4. Fitousis, K.; Klasek, R.; Mason, P.E.; Masud, F. Evaluation of a pharmacy managed heparin protocol for extracorporeal membrane oxygenation patients. **2017**, *32*, 238-244, doi:10.1177/0267659116678057.
5. Wilcox, C.; Etchill, E.; Giuliano, K.; Mayasi, Y.; Gusdon, A.M.; Cho, I.C.; Kim, B.S.; Bush, E.L.; Geocadin, R.G.; Whitman, G.J.; et al. Acute Brain Injury in Postcardiotomy Shock Treated With Venoarterial Extracorporeal Membrane Oxygenation. *Journal of cardiothoracic and vascular anesthesia* **2021**, *35*, 1989-1996, doi:10.1053/j.jvca.2021.01.037.
6. Raman, J.; Alimohamed, M.; Dobrilovic, N.; Lateef, O.; Aziz, S. A comparison of low and standard anti-coagulation regimens in extracorporeal membrane oxygenation. *The Journal of heart and lung transplantation : the official publication of the International Society for Heart Transplantation* **2019**, *38*, 433-439, doi:10.1016/j.healun.2019.01.1313.
7. Wood, K.L.; Ayers, B.; Gosev, I.; Kumar, N.; Melvin, A.L.; Barrus, B.; Prasad, S. Venoarterial-Extracorporeal Membrane Oxygenation Without Routine Systemic Anticoagulation Decreases Adverse Events. *The Annals of thoracic surgery* **2020**, *109*, 1458-1466, doi:10.1016/j.athoracsur.2019.08.040.
8. Marinoni, M.; Migliaccio, M.L.; Trapani, S.; Bonizzoli, M.; Gucci, L.; Cianchi, G.; Gallerini, A.; Tadini Buoninsegni, L.; Cramaro, A.; Valente, S.; et al. Cerebral microemboli detected by transcranial doppler in patients treated with extracorporeal membrane oxygenation. **2016**, *60*, 934-944, doi:https://doi.org/10.1111/aas.12736.
9. Lim, J.Y.; Kim, J.B.; Choo, S.J.; Chung, C.H.; Lee, J.W.; Jung, S.H. Anticoagulation During Extracorporeal Membrane Oxygenation; Nafamostat Mesilate Versus Heparin. *The Annals of thoracic surgery* **2016**, *102*, 534-539, doi:10.1016/j.athoracsur.2016.01.044.
10. Park, S.J.; Kim, J.B.; Jung, S.-H.; Choo, S.J.; Chung, C.H.; Lee, J.W. Outcomes of extracorporeal life support for low cardiac output syndrome after major cardiac surgery. *The Journal of thoracic and cardiovascular surgery* **2014**, *147*, 283-289, doi:10.1016/j.jtcvs.2012.11.006.
11. Laimoud, M.; Alanazi, M.; Qureshi, R. Hospital outcomes after emergent peripheral veno-arterial extracorporeal membrane oxygenation in adult patients presenting with cardiogenic shock. **2021**, *17*, 103-109, doi:10.22514/sv.2021.118.
12. Melehy, A.; Sanchez, J.; Fried, J.A.; Takeda, K. Prosthetic valve thrombosis during extracorporeal life support for postcardiotomy shock. *Interactive CardioVascular and Thoracic Surgery* **2020**, *31*, 573-575, doi:10.1093/icvts/ivaa125 %J Interactive CardioVascular and Thoracic Surgery.
13. Cooper, E.; Burns, J.; Retter, A.; Salt, G.; Camporota, L.; Meadows, C.I.; Langrish, C.C.; Wyncoll, D.; Glover, G.; Ioannou, N.; et al. Prevalence of Venous Thrombosis Following Venovenous Extracorporeal Membrane Oxygenation in Patients With Severe Respiratory Failure. *Critical care medicine* **2015**, *43*, e581-584, doi:10.1097/ccm.0000000000001277.
14. Brunet, J.; Valette, X.; Ivascau, C.; Lehoux, P.; Sauneuf, B.; Dalibert, Y.; Masson, R.; Sabatier, R.; Buklas, D.; Seguin, A.; et al. Extracorporeal Life Support for Refractory Cardiac Arrest or Shock: A 10-Year Study. *ASAIO journal (American Society for Artificial Internal Organs : 1992)* **2015**, *61*, 676-681, doi:10.1097/mat.0000000000000282.
15. Ruggeri, L.; Franco, A.; Alba, A.C.; Lembo, R.; Frassoni, S.; Scandroglio, A.M.; Calabrò, M.G.; Zangrillo, A.; Pappalardo, F. Coagulation Derangements in Patients With Refractory Cardiac Arrest Treated With Extracorporeal Cardiopulmonary Resuscitation. *Journal of cardiothoracic and vascular anesthesia* **2019**, *33*, 1877-1882, doi:https://doi.org/10.1053/j.jvca.2018.11.014.
16. Becher, P.M.; Goßling, A.; Schrage, B.; Twerenbold, R.; Fluschnik, N.; Seiffert, M.; Bernhardt, A.M.; Reichenspurner, H.; Blankenberg, S.; Westermann, D. Procedural volume and outcomes in patients undergoing VA-ECMO support. *Critical Care* **2020**, *24*, 291, doi:10.1186/s13054-020-03016-z.

17. Bidar, F.; Lancelot, A.; Lebreton, G.; Pineton de Chambrun, M.; Schmidt, M.; Hékimian, G.; Juvin, C.; Bréchot, N.; Schoell, T.; Leprince, P.; et al. Venous or arterial thromboses after venoarterial extracorporeal membrane oxygenation support: Frequency and risk factors. *The Journal of heart and lung transplantation : the official publication of the International Society for Heart Transplantation* **2021**, *40*, 307-315, doi:10.1016/j.healun.2020.12.007.
18. Saeed, O.; Jakobleff, W.A.; Forest, S.J.; Chinnadurai, T.; Mellas, N.; Rangasamy, S.; Xia, Y.; Madan, S.; Acharya, P.; Algodi, M.; et al. Hemolysis and Nonhemorrhagic Stroke During Venoarterial Extracorporeal Membrane Oxygenation. *The Annals of thoracic surgery* **2019**, *108*, 756-763, doi:10.1016/j.athoracsur.2019.03.030.
19. Le Guennec, L.; Cholet, C.; Huang, F.; Schmidt, M.; Bréchot, N.; Hékimian, G.; Besset, S.; Lebreton, G.; Nieszkowska, A.; Leprince, P.; et al. Ischemic and hemorrhagic brain injury during venoarterial-extracorporeal membrane oxygenation. *Annals of intensive care* **2018**, *8*, 129, doi:10.1186/s13613-018-0475-6.
20. Malfertheiner, M.V.; Koch, A.; Fisser, C.; Millar, J.E.; Maier, L.S.; Zeman, F.; Poschenrieder, F.; Lubnow, M.; Philipp, A.; Müller, T. Incidence of early intra-cranial bleeding and ischaemia in adult veno-arterial extracorporeal membrane oxygenation and extracorporeal cardiopulmonary resuscitation patients: a retrospective analysis of risk factors. **2020**, *35*, 8-17, doi:10.1177/0267659120907438.
21. Melehy, A.; Ning, Y.; Kurlansky, P.; Kaku, Y.; Fried, J.; Hastie, J.; Ciolek, A.; Brodie, D.; Eisenberger, A.B.; Sayer, G.; et al. Bleeding and Thrombotic Events During Extracorporeal Membrane Oxygenation for Postcardiotomy Shock. *The Annals of thoracic surgery* **2022**, *113*, 131-137, doi:10.1016/j.athoracsur.2021.02.008.
22. Moussa, M.D.; Soquet, J.; Lamer, A.; Labreuche, J.; Gantois, G.; Dupont, A.; Abou-Arab, O.; Rousse, N.; Liu, V.; Brandt, C.; et al. Evaluation of Anti-Activated Factor X Activity and Activated Partial Thromboplastin Time Relations and Their Association with Bleeding and Thrombosis during Veno-Arterial ECMO Support: A Retrospective Study. *Journal of clinical medicine* **2021**, *10*, doi:10.3390/jcm10102158.
